# Brain Tissue Low-Level Mosaicism for *MTOR* Mutation Causes Smith–Kingsmore Phenotype with Recurrent Hypoglycemia—A Novel Phenotype and a Further Proof for Testing of an Affected Tissue

**DOI:** 10.3390/diagnostics11071269

**Published:** 2021-07-15

**Authors:** Krzysztof Szczałuba, Małgorzata Rydzanicz, Anna Walczak, Joanna Kosińska, Agnieszka Koppolu, Anna Biernacka, Katarzyna Iwanicka-Pronicka, Wiesława Grajkowska, Elżbieta Jurkiewicz, Paweł Kowalczyk, Rafał Płoski

**Affiliations:** 1 Department of Medical Genetics, Medical University of Warsaw, Pawinskiego 3c Str., 02-106 Warsaw, Poland; mrydzanicz@wum.edu.pl (M.R.); walczak.m.anna@gmail.com (A.W.); joanna.kosinska@wum.edu.pl (J.K.); agnieszka.koppolu@gmail.com (A.K.); biernackann@gmail.com (A.B.); 2 Department of Audiology and Phoniatrics, The Children’s Memorial Health Institute, 04-730 Warsaw, Poland; k.iwanicka-pronicka@ipczd.pl; 3 Department of Pathology, The Children’s Memorial Health Institute, 04-730 Warsaw, Poland; w.grajkowska@ipczd.pl; 4 Department of Diagnostic Imaging, The Children’s Memorial Health Institute, 04-730 Warsaw, Poland; e-jurkiewicz@o2.pl; 5 Department of Neurosurgery, The Children’s Memorial Health Institute, 04-730 Warsaw, Poland; p.kowalczyk@ipczd.pl

**Keywords:** *MTOR*, mosaicism, hemimegalencephaly, metabolism, hypoglycemia, Smith–Kingsmore syndrome, whole exome sequencing

## Abstract

De novo somatic variants in genes encoding components of the PI3K–AKT3–mTOR pathway, including *MTOR*, have been linked to hemimegalencephaly or focal cortical dysplasia. Similarly to other malformations of cortical development, this condition presents with developmental delay and intractable epilepsy, often necessitating surgical treatment. We describe a first patient with the Smith–Kingsmore syndrome phenotype with recurrent hypoglycemia caused by low-level mosaic *MTOR* mutation restricted to the brain. We provide discussion on different aspects of somatic mosaicism. Deep exome sequencing combined with a variant search in multiple tissues and careful phenotyping may constitute a key to the diagnosis of the causes of rare brain anomalies.

## 1. Introduction

Uninterrupted PI3K–AKT3–mTOR signaling is essential for the maintenance of a balanced cell proliferation/differentiation rate [1,2,3,4]. Thus, mutations in any component of the pathway may lead to cancer or abnormal differentiation, which often affects neural progenitors resulting in congenital brain malformations. The *MTOR* gene encodes Mechanistic Target of Rapamycin Kinase protein, which provides instructions for cell growth, proliferation and survival. Pathogenic gain-of-function variants in *MTOR* have been linked to major malformations of cortical development (MCDs): focal cortical dysplasia (FCD) and hemimegalencephaly (HME) [5,6,7,8].

Syndromic hemimegalencephaly caused by *MTOR* mutations is part of the Smith–Kingsmore phenotype (SKS) (MIM#616638), consisting mainly of intellectual disability/developmental delay, seizures, macrocephaly, facial dysmorphism and patchy skin pigmentation defects [9,10]. The SKS eponym has been exclusively used for a disorder caused by germline or mosaic constitutive *MTOR* variants that are unequivocally present in all tissues [11].

Herein, we present the case of a Smith–Kingsmore syndrome phenotype caused by a known de novo mosaic *MTOR* mutation restricted to the brain tissue. The variant was not found in blood or hair bulbs, and it was present in only 8% of the tested brain tissue after surgery. The 5-year-old boy presented with severe developmental delay, intractable seizures, global hypotonia, hemimegalencephaly with pachygyria and polymicrogyria, streaks of hyper-/hypopigmentation, and a history of hypoglycemia. The striking feature of recurrent infantile hypoglycemia has never been reported previously in the literature, yet this is consistent with the PI3K–AKT3–mTOR pathway’s role in glucose utilization.

## 2. Materials and Methods

The study was conducted according to the guidelines of the Declaration of Helsinki. It was approved by the Ethical Committee at the Warsaw Medical University and CMHI.

DNA extracted from the brain tissue of the proband (enlarged left temporal lobe) was analyzed by whole-exome sequencing (WES), which was performed using SeqCap EZ MedExome (SureSelect Human All Exon V5, Agilent, Santa Clara, CA, USA) according to manufacturer’s instructions. Library was pair-end sequenced (2 × 100 bp) on HiSeq 1500 (Illumina, San Diego, CA, USA) to the mean depth of 80×; 92% of target bases were covered at a minimum of 20×, whereas 97% had coverage of min. 10×. Raw data were analyzed as previously described [12] with hg19 genomic build used for alignments. The analysis of WES data was performed as follows: after inspection for pathogenic variants described in HGMD, variants passing a default quality filter were filtered to include those with <1% minor allele frequency in gnomAD and an in-house database of >3000 exomes from Polish population. Synonymous and deep intronic/non-coding variants were excluded. The remaining variants were searched for potentially biallelic variants consistent with autosomal recessive inheritance and monoallelic variants potentially causative of dominantly inherited diseases (autosomal or X-linked).

Amplicon Deep Sequencing (ADS) for the selected *MTOR* variant was performed in the proband (brain tissue), his parents and DNA extracted from proband’s additional samples: hair follicles, peripheral blood and urine sediment using Nextera XT Kit (Illumina, San Diego, CA, USA). The variant was sequenced on HiSeq 1500 (Illumina, San Diego, CA, USA).

FASTQ and VCF files from WES are available on request to qualified researchers.

## 3. Case Presentation

### 3.1. Clinical and Pathology Report

A currently 5-year-old boy was born spontaneously at 40 weeks from an uncomplicated first pregnancy of young, unrelated, healthy and nonconsanguineous parents, with an Apgar score of 10 after 1 and 5 min. The birthweight was 2980 g (25 c), length 53 cm (90 c), and head circumference 36 cm (50–75 c). Hypoglycemia as low as 30 mg% ensued at 3 weeks of life, which was treated successfully with 10% glucose and frequent feeding with starch. The infant was gaining weight slowly due to swallowing problems and weakness of oral muscles. Epileptic seizures, particularly lower-limb clonic episodes, oculomotor attacks and oral automatisms started within the first month of life. Up to about 50 episodes a day were observed that were intractable to Convulex, Synacthen and Sabril, with a later addition of cannabis and levetiracetam (Keppra). EEG testing revealed hypsarrhythmia.

At 8 months, an antireflux operation and PEG tube insertion were performed due to severe feeding problems. Further episodes of hypoglycemia of <30–40 mg% were noted that were not due to metabolic cause, nor were they hyperinsulinemic. Thus, a hypothesis of an abnormal utilization of glucose within the CNS, as present in a disturbed PI3K–AKT3–MTOR pathway, was put forward.

**Figure 1:**
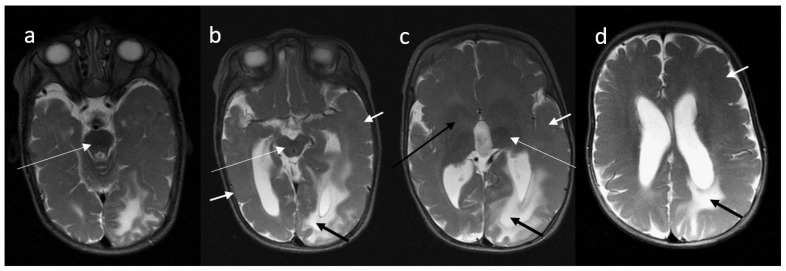
A 9-month-old boy, MR brain examination, (**a**–**d**)—axial T2-weighted images. T2-weighted images in axial plane demonstrate asymmetry of the cerebral hemispheres with the left one slightly larger than right. Diffuse loss of sulcation, broad gyri in association with diffuse polymicrogyria/pachygyria and indistinct gray–white matter differentiation are seen in both cerebral hemispheres (short white arrows on (**b**–**d**)). Lack of white matter myelination: weak myelination is visible only in posterior limbs of the internal capsules (long white arrow on (**c**)) and brainstem (long white arrows on (**a**,**b**)) and cerebellar peduncles (not shown). Basal ganglia show abnormal appearance bilaterally (long black arrow on (**c**)). The white matter of the left occipital lobe and posterior part of left parietal and frontal lobes is abnormally hyperintense on T2-WI (short black arrows on (**b**–**d**)). Brain stem (long white arrows on (**a**,**b**)) and splenium of the corpus callosum are hypoplastic. The lateral ventricles and third ventricle are enlarged.

A brain MRI scan performed at the age of 9 months revealed left-sided hemimegalencephaly, generalized cortical dysplasia with polymicrogyria and pachygyria, hypoplasia of the midbrain and brainstem, and delayed myelination (Figure 1).

At the age of 1.5 years, the boy underwent a two-stage quadrantectomy of the left temporo-occipito-parietal area. Despite the procedure, seizures did not resolve. On anticonvulsant treatment with levetiracetam (Keppra) and lamotrigine (Lamitrin), over 30 episodes a day were observed. A tracheostomy tube was inserted following surgery.

At 5 years, the boy’s psychomotor development is severely delayed. He is unable to lift his head up, nor can he sit supported or stand with assistance. He cannot speak a single word. His physical parameters are: height 97 cm (<3 c), weight 13 kg (<3 c) and head circumference 52 cm (75–90 c).

The examination shows severe global hypotonia, weak spontaneous activity, elongated skull, prominent forehead, dysplastic ears, bilateral hip dislocation, knee and elbow contractures, and streaks of hyperpigmentation in the lower limbs following the pattern of Blaschko lines. Despite the analgesic treatment, the child constantly shows features of chronic pain. He is fed through a PEG tube and is on a no-sugar, no-dairy and no-gluten diet.

Due to a high suspicion of PI3K–AKT3–MTOR pathway disorder and intractable epilepsy diagnosis, rapamycin treatment was initiated but discontinued shortly thereafter due to decreased immunity and increasing frequency of seizures.

Additional investigations revealed vision loss that was confirmed with VEP testing. Cortical reaction to light was not detected. The results of the objective hearing tests were normal. There were no other congenital anomalies on extensive internal organ imaging.

**Figure 2:**
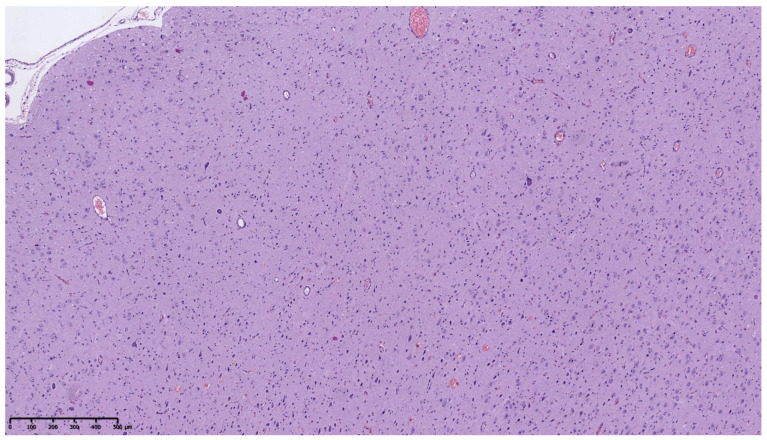
Hematoxylin and eosin staining (HE). Disorganized cytoarchitecture of cerebral cortex (100×).

Histopathological analysis of the brain tissue obtained from the temporal lobe of the enlarged left hemisphere revealed a widened, abnormal cortex ribbon without demarcation from subcortical white matter. The complete loss of horizontal cortical lamination was observed (Figure 2).

**Figure 3:**
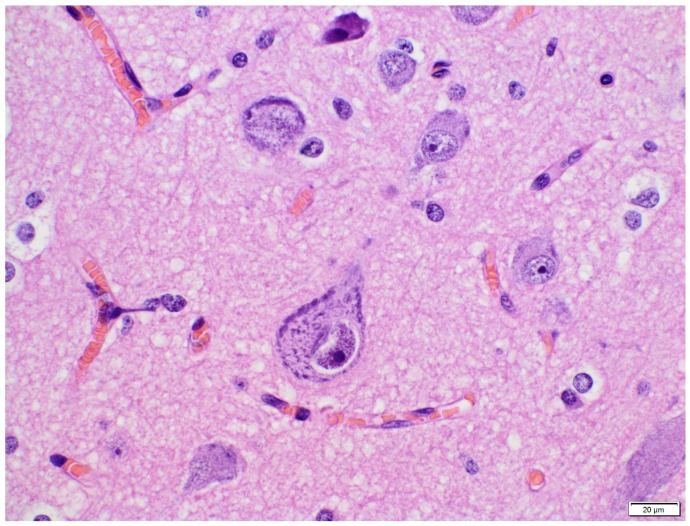
HE. Cytomegalic and dysmorphic neurons (600×).

Neuronal density was reduced. Particularly striking was the presence of the cytological abnormalities of neurons with neuronal cytomegaly (Figure 3).

**Figure 4:**
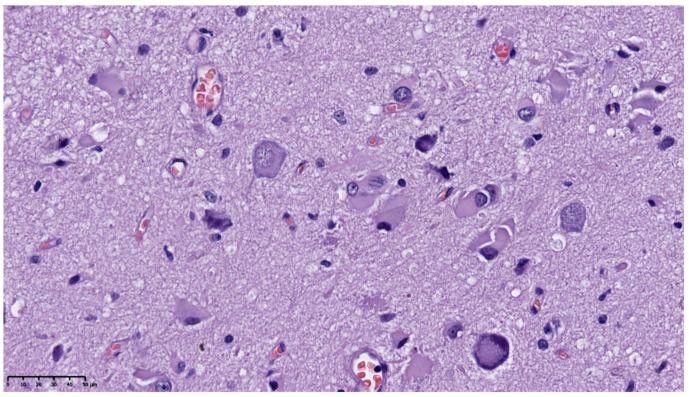
HE. Dysmorphic neurons and balloon cells (400×).

There were abnormally large neurons, often bizarrely shaped with multilobulated nuclei. Dysmorphic neurons and some balloon cells were present as well (Figure 4).

**Figure 5:**
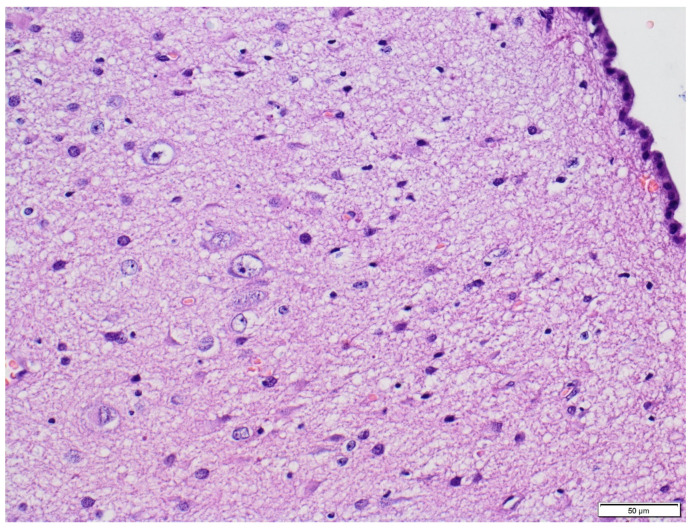
HE. Abnormal neurons present ectopically in white matter (400×).

The abnormal neurons were scattered throughout the cortex and present ectopically in white matter (Figure 5).

**Figure 6:**
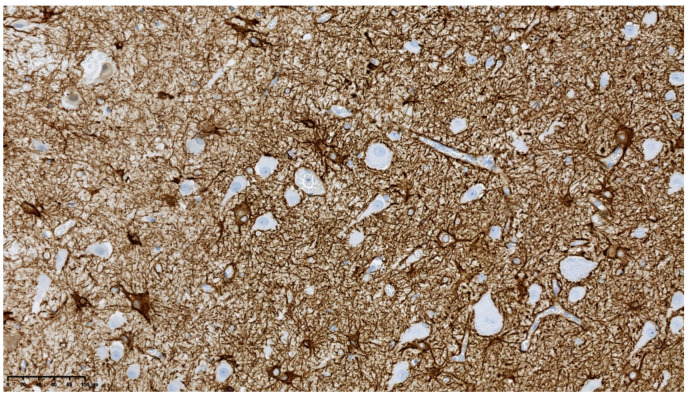
GFAP immunoreactivity in reactive astrocytes (200×).

Reactive gliosis was observed (Figure 6).

**Figure 7:**
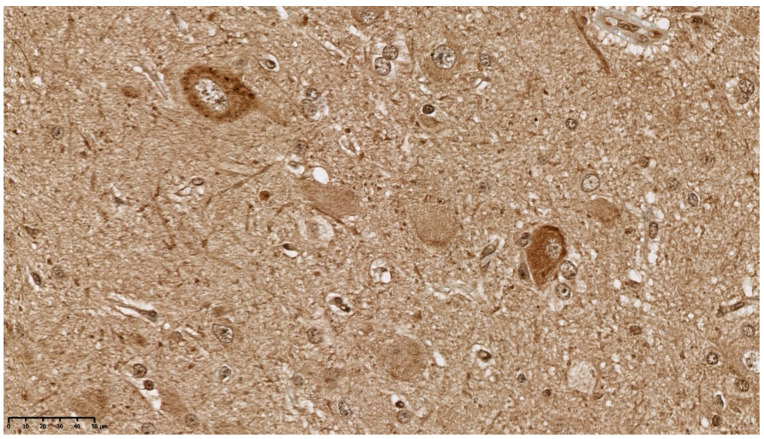
Immunoreactivity for mTOR kinase protein in enlarged neurons (400×).

The pathological diagnosis of hemimegalencephaly (HME) was established. In some enlarged neurons strong immunoreactivity for mTOR kinase protein was detected (Figure 7).

### 3.2. Genetic Report

**Figure 8:**
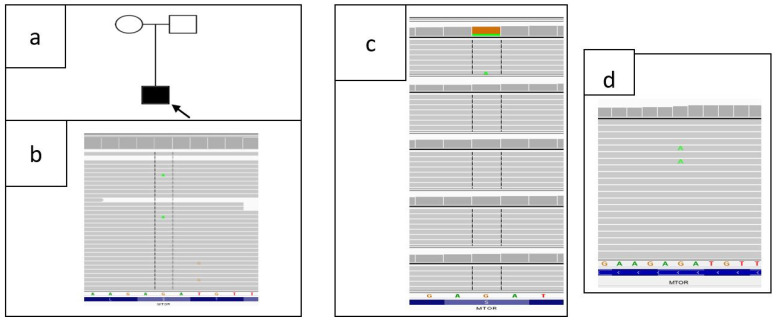
(**a**) Pedigree of proband’s family: proband is marked with black arrow; (**b**) WES results of fragment of MTOR in proband’s DNA, obtained from brain tissue, using Integrative Genomic Viewer (IGV)—variant count 9/116 (8%); (**c**) Results from sequencing of DNA obtained from proband’s peripheral blood sample: amplicon deep sequencing (ADS)—(1) brain tissue 787/3420 (23%) (proband); (2) blood: 11/5862 (0%); (3) hair follicles: 11/4132 (0%); mother (5/2461) and father (7/2852)—DNA from peripheral blood; (**d**) ADS results of urine sediment 6/242 (2%).

WES in brain post-surgical tissue (enlarged left temporal lobe) revealed an *MTOR* variant (hg19; g.chr1:011184573-G>A, NM_004958.4:c.6644C>T; p.(Ser2215Phe)) in 8% (9/116 reads) of brain tissue, which was deemed consistent with the proband’s phenotype. The selected variant was further validated in the proband (brain tissue), his parents and DNA extracted from the proband’s additional samples: hair follicles, peripheral blood and urine sediment by amplicon deep sequencing (ADS) performed using Nextera XT Kit (Illumina, San Diego, CA, USA) and sequenced on HiSeq 1500 (Illumina, San Diego, CA, USA). The p.(Ser2215Phe) was absent in DNA samples extracted from peripheral blood or hair follicles of the proband (11/5862 and 11/4132 reads were analyzed, respectively), while in the brain tissue its level equaled 23% (787/3420 reads) (Figure 8).

ADS sequencing of urine sediment DNA isolate revealed 2% (6/242) of variant c.6644C>T in *MTOR*. It cannot be excluded that with such low coverage and only six reads with an altered allele the result may indicate a sequencing artifact. Parental studies showed the absence of the variant in the proband’s mother’s and father’s blood sample. The p.(Ser2215Phe) was absent in all tested databases, including an in-house database of >3000 WES of Polish individuals. Detected variant p.(Ser2215Phe) was previously described as a somatic mutation causing focal cortical dysplasia by Møller et al., Mirzaa et al., Nakashima et al., Lim et al., and Leventer et al. [7,8,9,13,14]. The clinical significance of detected variant p.(Ser2215Phe) is determined as likely pathogenic/pathogenic by the ClinVar database (https://www.ncbi.nlm.nih.gov/clinvar/variation/156703/ accessed on 13 July 2021). Additionally, the variant was predicted as damaging by all used programs (MutationAssessor, MetaSVM, MetalR, Provean, LRT, SIFT, DANN; https://varsome.com accessed on 13 July 2021).

## 4. Discussion

Clinical effects of *MTOR* mutations were analyzed by Mirzaa et al. [7], who first divided them according to resulting brain malformations into focal cortical dysplasia (FCD), asymmetric megalencephaly (hemimegalencephaly, HMEG) with polymicrogyria and cutaneous markings, and diffuse megalencephaly (MEG). It was noted that certain *MTOR* variants predominate in each group, but there is also a difference in the distribution of mutations among the tested tissues. Thus, the FCD phenotype is caused by somatic alterations detected in abnormal tissue post-surgery, but not in blood or saliva [7,8,9,13,14]. In FCD, the mutation burden in affected tissue may be as low as 1.5% and still causative [8,13]. For the two other mentioned phenotypes, where brain tissue may be unavailable due to the lack of indications for surgery, the mutation in mosaic or non-mosaic form is seen in white blood cells, skin fibroblasts or saliva [7]. Lastly, if all the tissues retain at least some degree of mosaicism for an *MTOR* variant, the term Smith–Kingsmore syndrome (SKS)(OMIM:616638) is used [11]. Gordo et al. (2018) summed up the main (found at least 50% of the time) features of SKS: intellectual disability, macrocephaly/megalencephaly, seizures, brain anomalies other than HME, curly/wavy hair, and ventriculomegaly. According to the authors, other symptoms frequently present are macrosomia at birth or specific dysmorphism.

In our patient, a severe developmental delay, epilepsy, complex brain anomalies (HME, ventriculomegaly, brain stem hypoplasia) and facial dysmorphism were noted. Thus, the boy clinically resembled the SKS phenotype, but of the four tested tissues the variant was found only in the brain (urine sample had a relatively low coverage).

A significant overlap exists among all the *MTOR* phenotypes. Mosaic variant p.Thr1977Ile in Mirzaa et al.’s study resulted in HME, polymicrogyria, seizures, hydrocephalus and cutaneous pigmentary mosaicism of Blaschko type [7]. This variant may be present in a mosaic state in skin fibroblasts derived from hyperpigmented streaks and be absent from blood, which is crucial in cases where brain tissue is not accessible [15]. Additionally, patient HME-1563 of Lee et al. with c.4448C>T variant presented with hemimegalencephaly and similar skin markings [16]. In this case, the mutation was observed only within the brain.

Pathogenic variant p.Ser2215Phe present in our proband was thus far seen only in the brain tissue of focal cortical dysplasia (FCD) individuals presenting exclusively with seizures [8,9]. In these patients, a very low level of mosaicism for the mutation (1–6%) restricted to the dysplastic focus was present. As shown in our example, a low-level 8% mosaicism in WES (23% in ADS) for p.Ser2215Phe already results in a complex SKS-like phenotype. A brain-only mutation burden of 9.7% may, in fact, be enough to cause extra-neuronal symptoms, as in the HME-1563 individual [16].

Nonetheless, the overall mutation burden in brain mosaics may not be a feasible factor in establishing a clinical course of the disorder. Instead, a detailed analysis of the alternate allele fraction in certain regions of the resected tissue may identify a crucial gradient of low-level mosaicism. In the only example of a patient analyzed in this way (LR13-389; p.Ser2215Phe), some areas of cortical dysplasia were devoid of mutation [7]. Interestingly, the presence of mutation did not seem to correlate with seizure focus [7]. In our case, we were not able to distinguish a certain pattern of mutation burden in affected brain tissue.

An intriguing and thus unreported finding in our patient with *MTOR* mutation was hypoglycemia that ensued in the neonatal period and was at that time successfully treated with 10% glucose. It would later recur at least twice at ages 8 and 13 months. The typical metabolic and hyperinsulinemic causes of hypoglycemia in our patient were excluded and it was pointed out that asymmetric brain growth may be linked to the higher glucose utilization by the tissues. Currently, hypoglycemia has been established as part of the phenotypic spectrum of mutations within five genes that belong to the PI3K–AKT–mTOR pathway: *AKT2, AKT3, PIK3CA, PIK3R2* and *CCND2A* [17,18,19,20,21]. Variants in *AKT2* and *AKT3* that are upstream of *MTOR* are activating variants, similarly to the gain-of-function *MTOR* mutation in our patient. The finding of hypoglycemia in our patient may have important implications for the management. Increasing the frequency of and/or the caloric load of enteral feeds could be the best way to treat it, while ensuing seizures may respond to a ketogenic diet [22].

## 5. Conclusions

Our report provides evidence for brain-only low-level *MTOR* mosaicism as causative of the Smith–Kingsmore syndrome phenotype. Moreover, it is the first to associate gain-of-function mutation in *MTOR* with hypoglycemia, which necessitates significant changes in therapeutic management for an affected child. For rare variants in PI3K–AKT–mTOR pathway genes, exome data should be carefully analyzed for the possibility of somatic mosaicism.

## Data Availability

The authors confirm that the data supporting the findings of this study are available within the article. Raw data were generated at the Department of Medical Genetics, Medical University of Warsaw. Derived data supporting the findings of this study are available from the corresponding authors (KS & RP) on request.
